# Piezo channels in the urinary system

**DOI:** 10.1038/s12276-022-00777-1

**Published:** 2022-06-14

**Authors:** Xu Li, Junwei Hu, Xuedan Zhao, Juanjuan Li, Yuelai Chen

**Affiliations:** Longhua Hospital, Shanghai University of Traditional Chinese Medicine, Shanghai, 200032 China

**Keywords:** Bladder disease, Bladder cancer, Prostate cancer, Oncogenesis

## Abstract

The Piezo channel family, including Piezo1 and Piezo2, includes essential mechanosensitive transduction molecules in mammals. Functioning in the conversion of mechanical signals to biological signals to regulate a plethora of physiological processes, Piezo channels, which have a unique homotrimeric three-blade propeller-shaped structure, utilize a cap-motion and plug-and-latch mechanism to gate their ion-conducting pathways. Piezo channels have a wide range of biological roles in various human systems, both in vitro and in vivo. Currently, there is a lack of comprehensive understanding of their antagonists and agonists, and therefore further investigation is needed. Remarkably, increasingly compelling evidence demonstrates that Piezo channel function in the urinary system is important. This review article systematically summarizes the existing evidence of the importance of Piezo channels, including protein structure, mechanogating mechanisms, and pharmacological characteristics, with a particular focus on their physiological and pathophysiological roles in the urinary system. Collectively, this review aims to provide a direction for future clinical applications in urinary system diseases.

## Urinary system disease: Understanding transmembrane protein family may increase treatment options

Extensive research into the complex roles of a family of transmembrane proteins present in the urinary system could lead to novel therapeutics for urinary system diseases. Recent research highlights the importance of piezo channel proteins in the urinary system. Piezo channels are a form of mechanically sensitive ion channel, which converts mechanical signals into biological signals. Xu Li and co-workers at Shanghai University of Traditional Chinese Medicine, China, reviewed piezo channel function and activity in the urinary system. Considerable insights have been gained into the physical structure and functions of Piezo1 and Piezo2 proteins which each respond to different mechanical stimuli and contribute in different ways to urinary system homeostasis. Mutations and disruption to these proteins may trigger diseases including cancer, but further investigations are needed to fully understand their mechanisms of aśction.

## Introduction

Mechanosensitive ion channels (MSCs) convert mechanical signals into electrochemical signals, which are essential for almost all mammalian cells^1^. The urinary system is important in the regulation of water and salt metabolism, the maintenance of acid-base balance, and hormone production^2–4^. As key mechanotransducers, MSCs are widely present in the urinary system in response to mechanical stimuli, including shear stress, bladder wall stretching, and urine flow^1,5–10^, resulting in a wide range of biological effects. Given the crucial physiological and pathological roles of MSCs in the urinary system, revealing their potential mechanotransduction mechanisms and characteristics as new targets for disease management is imperative.

Since the discovery of MSCs by Corey and Hudspeth in their analysis of the ionic basis of the receptor potential in a vertebrate hair cell in 1979, which led to the first recording of electrical currents activated by mechanical force, studies exploring these types of ion channels have emerged^11,12^. However, the identification criteria of MSCs have long been controversial. In 2007, Christensen AP et al. summarized the existing research and proposed a series of criteria for identifying candidate channels as MSCs, which facilitated the development of this field^13^. In 2010, researchers in the Ardem Patapoutian laboratory made major progress in the investigation of MSCs^14^. In particular, a glass microprobe was used to apply force to the surface of the candidate cells, and the current model of the cells was recorded via patch-clamp^14^. It was found that the Neuro2A (N2A) mouse neuroblastoma cell line expressed stable and sustained mechanically activated (MA) currents^14^. Then, we tested candidate genes in N2A cells with siRNA knockdown, discovered the existence of Piezo, and confirmed that there are two members of the Piezo family, Piezo1 and Piezo2^14^. These molecules are a type of evolutionarily conserved transmembrane (TM) protein with little sequence homology to other known ion channel families^14^. For over a decade, since the discovery of Piezo channels, a substantial number of related studies have been published that have extensively elucidated the physiopathology related to these molecules. Moreover, Piezo channels are expected to become a new target for drug discovery. Therefore, the Nobel Prize in Physiology or Medicine 2021 was awarded to Ardem Patapoutian in recognition of his outstanding contribution to the field of MSCs.

In this review, we summarize the data on Piezo channels, including their structure, mechanogating mechanisms, pharmacological properties, and physiological functions in human body systems. By emphasizing the known physiological and pathophysiological roles of Piezo channels in the urinary system, we highlight the potential implications of targeting this cation channel family for therapy to expand the horizons for the treatment of urinary system diseases.

## Overview of piezo channels

### Structure of Piezo channels

#### Piezo1

**Fig. 1:**
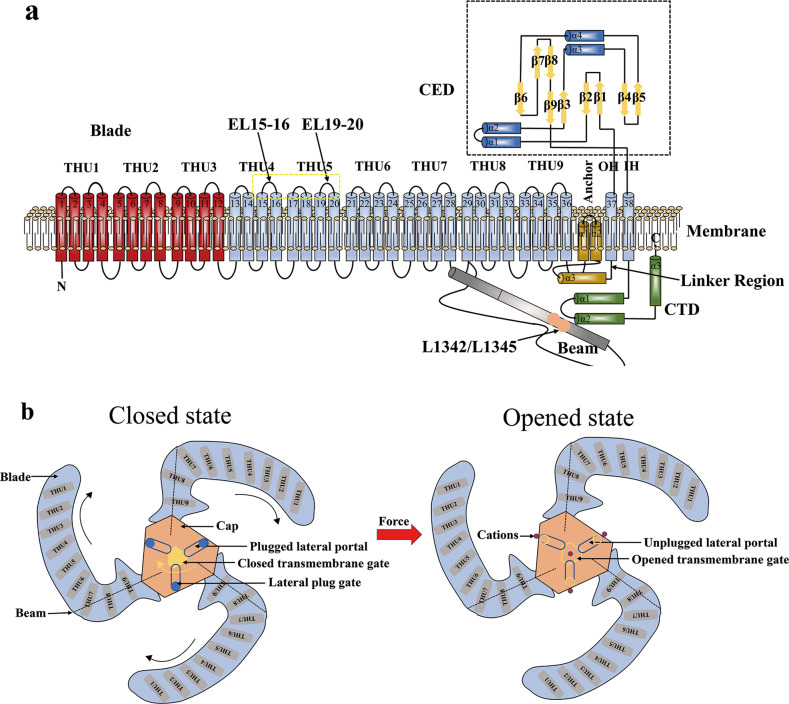
The 38-TM topology model, and mechanogating mechanisms from the extracellular view of the mouse Piezo1 channel (adapted from 15, 23). **a** A 38-TM topology structure of Piezo1. The dark red THU1—THU3 indicates unresolved areas. The linker region is the key area in which SERCA2 suppresses the MA current. The yellow dashed box is the extracellular loops EL15-16 and EL19-20, which are the key mechanotransduction sites for the hydrophilic small molecule agonist Jedi. The beam might utilize two leucine residues, L1342/L1345, as a pivot to transfer the fine mechanical force felt by the blade to the central ion-conducting pore module through a lever-like mechanism and thus selectively expose the cations. Three sets of such topologies are assembled into Piezo1, which possesses a total of 114 TM helices, making it one of the most complex mechanosensors. **b** Each blade twists in a clockwise direction to form a propeller-like structure from the extracellular view. Under the stimulation of mechanical force, the clockwise movement of the cap might open the TM gate. Through the leverage of the beam, the lateral plug gate might be partially unplugged, and the lateral portal might be opened, thus allowing cations to flow in.

Analyzing structure is the key to understanding functionality. Due to advancements in cryoelectron microscopy (cryo-EM) and X-ray crystallography, the high-resolution three-dimensional structure of the Piezo1 channel has been clearly presented^15–17^. With the trimeric three-blade, propeller-like medium-resolution cryo-EM structure of the mouse Piezo1 channel^18^, many pieces of information about TM regions were illuminated^15–17^ (Fig. 1), promoting understanding of the structure-function relationship. The mouse Piezo1 channel consists of three modules containing 2547 residues^18–20^. The central ion-conducting pore module is responsible for ion permeability and selectivity and includes the outer helices (OHs), inner helices (IHs), intracellular C-terminal domains (CTDs), and extracellular C-terminal domains (CEDs)^18–20^. The mechanosensing module includes peripheral propeller blades^18–20^. The mechanotransduction module consists of 90 Å long intracellular beams, anchors, and CTDs^18–20^ (Fig. 1a). Three CEDs form an extracellular cap structure above the OHs and IHs^15,18^. A single blade contains nine repeating transmembrane helical units (THUs) comprising four TM helices each^15,16,19^ (Fig. 1a). Together with OH and IH, these structures form a highly curved, nonplanar arrangement 38-TM topology^15,16,19^ (Fig. 1). The joint linking the three groups of THUs (THU7-THU9) and the adjacent three groups of THUs (THU4-THU6) is a physical bend, which is approximately 100 degrees from the extracellular view^15^ (Fig. 1b). Importantly, the conformation of this unusually curved blade structure might change with the tension and curvature of the membrane^15,19^. Between the intracellular surface of THU7-THU9 and CTD, the beam structure connecting TM28 and the central ion-conducting pore was identified^15–18^ (Fig. 1). It is possible that this structure utilizes two leucine residues, L1342/L1345, as the pivot to transfer the fine mechanical force on the blades to the central ion-conducting pore module through a lever-like mechanism, thereby selectively exposing the cations^15,21^.

#### Piezo2

**Fig. 2:**
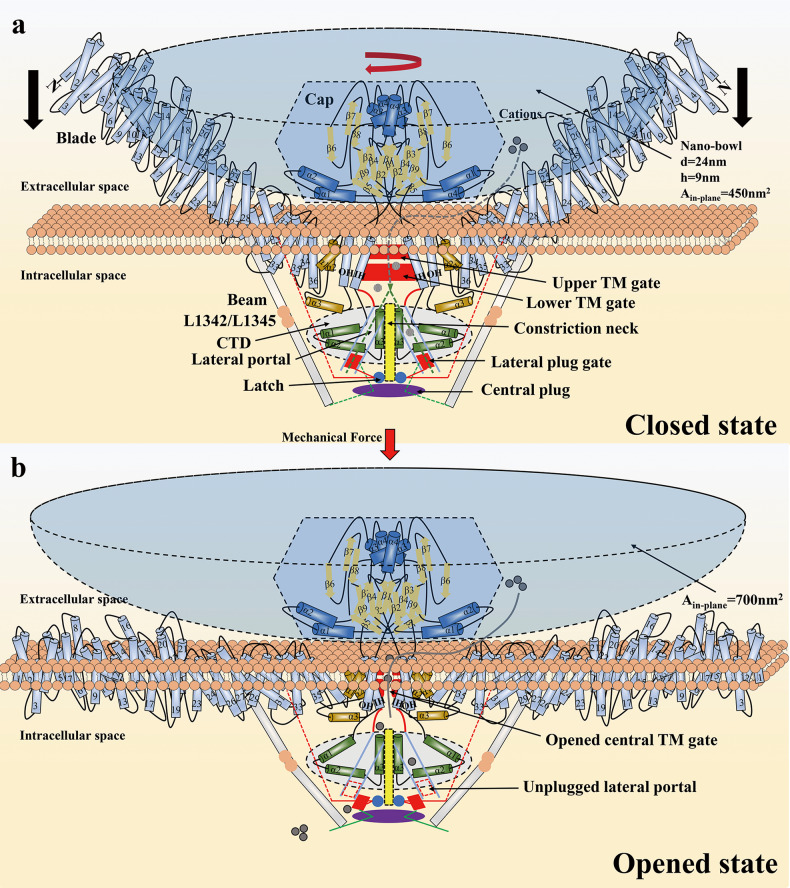
Structure and mechanogating mechanisms of the mouse Piezo2 channel. **a** Piezo2 channel in the closed state. The clockwise movement of the cap might control the opening of the extracellular fenestration sites and allows the penetration of cations. The cyan nanobowl structure is located in a three-dimensional space formed by three highly curved blades. The yellow structure is the constriction neck, and it is not clear whether it is part of the ion-conducting path. The green dashed line represents the ion-conducting routes where cations enter the central pore from the extracellular fenestration sites and then flow in the intracellular space through three lateral portals. The black arrow indicates that under mechanical stimulation, the blades move toward the plasma membrane and tend to be flat. **b** The nanobowl also changes as the blades become completely flat. The in-plane membrane area might expand from 450 nm2 in the closed state to 700 nm2 in the open state, which might be the structural basis for the mechanosensitivity of Piezo channels. At the same time, the mechanical force transmitted by the beam might unplug the lateral plug gates, contributing to opening the intracellular ion conduction routes so that the cations can enter.

Structurally, Piezo2 has approximately 42% sequence homology to Piezo1 in the cryo-EM three-dimensional structure^14,22^. Piezo1/2 is a homotrimer assembled from three blades with a total of 114 TM helices^15,22^. This finding not only indicates that Piezo channels are the membrane proteins with the largest number of transmembrane passes discovered thus far but also suggests that they might mediate mechanotransduction functions by similar mechanogating mechanisms^22^. The completely resolved Piezo2 structure shows that under the interaction of the hydrophobic belts formed by TM helices, three blades curved into a nanobowl configuration with a midplane opening diameter of 24 nm and a depth of 9 nm, which fully wrapped the extracellular cap^22,23^ (Fig. 2a). Nanobowls are potentially the structural basis for Piezo channels to be highly mechanosensitive^22,23^. The distal and proximal ends of the beam are connected to the clasp and latch, respectively, by hydrogen bonds, which are the key structures for the lever-like transduction of the beam^19,21,22^. The highly conserved IHs in the Piezo family are an integral part of the central pore module^22^. Of interest, in contrast to Piezo1, the two transmembrane constriction sites (L2743, F2754 and E2757) in Piezo2 IHs indicate that the central pore is relatively narrow^15,22^. Therefore, it is postulated that the two constriction sites might be the upper and lower transmembrane gates, controlled by the upward and clockwise movement of the cap^22–24^ (Fig. 2). In addition, three lateral portals were found in the central pores of Piezo1 and Piezo2^15,22^. Mutating residues of lateral portals will alter the ion permeability, indicating that they are most likely part of the ion-conducting pathway^22^. In the cytoplasmic region of the central pore module, there is a 10 Å long constriction neck consisting of three amino acid residues^22^ (Fig. 2a). This structure has also been identified in Piezo1, yet whether it is part of the ion-conducting pathway is unclear^22,25^.

### Mechanogating mechanisms of Piezo channels

Considering the important physiological and pathological functions of Piezo channels and their potential as new pharmacological targets for the future treatment of diseases, exploring the mechanogating mechanisms of Piezo channels is particularly crucial. A 14-residue intracellular linker sequence between the anchor and OHs is a key region where Piezo1 interacts with sarcoplasmic/endoplasmic-reticulum Ca^2+^ ATPase 2 (SERCA2)^26^ (Fig. 1a). The aforementioned findings suggest that Piezo1 utilizes peripheral blades to gate the central pore module^26^. These discoveries regarding the linker region provide a direction for uncovering the ingenious mechanogating and chemical regulatory mechanisms of Piezo channels.

#### Lever-like mechanogating mechanism

The proposal of the lever-like mechanogating mechanism of Piezo channels is important for understanding the basic life process of the conversion of mechanical stimulation into electrical signals^15,21,22^. The blades, composed of THUs, sense stimulation from mechanical forces or biomolecules, and the beam-constituted lever-like device transmits mechanical or chemical signals to the central pore module, further opening the intracellular lateral pathways^19,23,27^ (Fig. 2b). THU1-6 cells at the end of the blades are highly curved; consequently, the presence of the lever-like apparatus is also conducive to responding to large-scale changes in membrane curvature^21^.

#### Cap-motion mechanogating mechanism

The cap structure, a homotrimer formed by CEDs, is located on top of the central pore module, which controls the opening of the extracellular fenestration sites and allows the penetration of cations^22,24^ (Fig. 1b and 2). Compared with Piezo2, the cap of Piezo1 has obvious clockwise movement^22^. Deleting the α1 and α2 helical structures of the cap in both Piezo1 and Piezo2 completely eliminated the whole-cell currents caused by mechanical stimulation, indicating that cap motion controls the opening of the upper and lower transmembrane gates^22^.

#### Plug-and-Latch mechanogating mechanism

Geng J et al. confirmed the conjecture that the three intracellular lateral portals of Piezo channels are cation-permeating entrances^15,22,25^ and identified the “lateral plug” domain that physically blocks the lateral ion-conducting routes and the “latch” structure that controls the lateral plug^25^ (Fig. 2a). Initially, the nine residues of the lateral pathways were subjected to combined site-directed mutation, and the permeability of Cl^−^ over Na^+^ (pCl/pNa) of the Piezo1 mutants was significantly increased. When all nine residues were mutated to lysine residues, the Piezo1 mutant became a completely anion-selective channel^25^, suggesting that the Piezo1 channel employs three lateral portals to allow permeation of cations. Subsequently, the RNA sequencing data of Piezo1 obtained with the Trinity method showed that it undergoes reassembly, and the spliced variant Piezo1.1 was identified^25^. Intriguingly, Piezo1.1 lacks an exon that encodes the lateral plug structure, suggesting that Piezo1 utilizes the alternative splicing mechanism for the expression of this key exon^25^. Additionally, further studies were carried out on Piezo1.1. Compared with Piezo1, Piezo1.1 has greater single-channel conductance, lower Ca^2+^ permeability, and greater mechanosensitivity^25^. In brief, the lateral plug structure affects the ion permeability and mechanosensitivity of Piezo1. Therefore, an ingenious plug-and-latch mechanogating mechanism was developed. When the mechanical force acts on the blade, the three lateral plugs are anchored to the intracellular central axis under the leverage of the beam so that the physical obstruction is lifted and the lateral pathways are opened, allowing cations to flow in^15,21,22,25^ (Fig. 2).

#### A dual mechanogating mechanism

A dual mechanogating mechanism that can be used to explain the complex gating system of Piezo channels has been proposed, in which the transmembrane gates are gated by cap motion, and the intracellular routes are controlled by a series of key elements of the blade-beam-plug-latch through a lever-like mechanism and a plug and latch mechanism^15,21,22,25,27^ (Fig. 2). Exploring the delicate mechanogating mechanisms of Piezo channels not only allows us to better understand how the Piezo family exerts biological effects but also, more importantly, will help us to use it as a new effective target for related drug development.

### Biophysical properties of Piezo channels

#### Inactivation

Piezo1 responds to various forms of mechanical stimuli, including poking, stretching, shear stress, and changes in local membrane tension^14,28,29^. In contrast, Piezo2 is less responsive to changes in stretching and local membrane tension^30–32^. Despite their differences, both Piezo1 and Piezo2 nonselectively allow the penetration of cations in the following order: Ca^2+^> K^+^ > Na^+^> Mg^2+^. They also generate an MA current^14^. In addition to showing different sensitivities to specific mechanical stimuli, Piezo1 and Piezo2 also have different inactivation characteristics, with significant voltage dependence^14,32,33^. Specifically, Piezo2 exhibits faster inactivation kinetics than Piezo1, which slows down with depolarization^14,33^. Site-directed mutagenesis of residues L2475 and V2476 in the IH and residues M2493/F2494 in the CTD completely eliminated the inactivation of Piezo1^34–36^. Intriguingly, the mutation of M2493/F2494 has no effect on the inactivation of Piezo2^34^, indicating that the inactivation mechanisms of Piezo2 and Piezo1 are not the same, which might be an inherent reason for their different inactivation kinetics. However, the inactivation of Piezo channels is not irreversible. In the case of depolarization resulting in outward permeation, almost all Piezo channels can be rescued from the inactivation caused by repeated mechanical stimuli^32^. The finding that pressure stimuli under positive potential conditions can transform Piezo1 into a pure voltage-gated channel was surprising^32^. Repeated inactivation and renaturation are essential features of Piezo channels, and whether they have biological significance regarding certain physiological and pathological aspects warrants further exploration. Furthermore, the altered inactivation kinetics of Piezo channels can lead to a variety of genetic diseases. Xerocytosis is caused by mutations that slow the inactivation and subsequent calcium ion flux of the Piezo1 channel^37^. Mutations in Piezo2 can cause a subtype of distal arthrogryposis^38^. These changes might cause pathology by accelerating the recovery of Piezo2 channels from inactivation^38^. Inactivation kinetics, as the pore characteristics of ion channels, show variable performance on Piezos. Therefore, many studies are still needed to explain the specific inactivation mechanism of Piezo channels.

#### Pharmacological regulation

Various Piezo channel blockers have been investigated recently. The small molecules ruthenium red and gadolinium can nonspecifically block Piezo channels^39^. The peptide toxin grammostola spatulate mechanotoxin 4 (GsMTx4) reduces the membrane tension near the Piezo1 channel by inserting into the lipid bilayer, consequently exerting a nonspecific inhibitory effect^40,41^. In addition, polycystin-2 (PC2)^42^ and SERCA^26^ are a class of proteins that can interact with Piezo1 and weaken the MA current by binding directly. Recently, the potential of the dietary fatty acid margaric acid (MA) to act on the beam structure to increase the mechanical force threshold level of Piezo2 channels has been shown to counteract the Piezo2 hypersensitivity caused by bradykinin^43^. This finding might help alleviate tactile allodynia caused by inflammation. In summary, the lack of Piezo channel-selective blockers is a roadblock in our understanding of their specific roles in physiological processes. Thus, further research on the pharmacology of Piezo channels is needed.

Currently, there are two known Piezo1 agonists, Yoda1 and Jedi1/2. Both agonists have been suggested to lower the mechanical force threshold of Piezo1, prolong the inactivation time and increase its mechanosensitivity^21,44^. The key site for the interaction between Yoda1 and Piezo1 is at the proximal end of the blade (residues 1961-2063)^45^. After binding, Yoda1 promotes membrane tension-induced blade motion through a wedge-like effect^45,46^. Additionally, Yoda1’s analog Dooku1^47^ and the traditional Chinese medicine extract Tbemoside A^48^ can competitively neutralize the agonistic effect of Yoda1. Notably, both Jedi1/2 and Yoda1 interact with the blade, but the activation of the Piezo1 channel by Jedi1/2 is faster, more reversible and less persistent, suggesting a different activation mechanism from Yoda1^21,45^. Consistent with the previous conclusions, Piezo1 becomes insensitive to Yoda1 and Jedi by mutating the beam pivot residues L1342/L1345. Accordingly, the beams are indispensable for downstream transduction pathways^21^. Recent studies have shown that fecal ssRNA might function as a natural ligand of Piezo1 to promote gastrointestinal chromaffin cells to secrete 5-HT, contributing to regulating the homeostasis of bones and intestines^49^. Together, these studies provide new insights for the treatment of bone and intestinal diseases. Similarly, the ceramide released by the sphingomyelinase subtype SMPD3 can change the local lipid microenvironment of Piezo1 and preclude its inactivation^50^. This process might have useful physiological effects. However, reports on specific activators of the Piezo2 channel are lacking, which substantially limits the current research. With continuous research on mechanogating mechanisms and pharmacological properties, the corresponding drug development will also usher in a new stage of treatment.

### Physiological roles of Piezo channels

Piezo channels play a vital biological role in mechanotransduction in varying mammalian cell types and are associated with a variety of physiological states. Mutations in Piezos pertinent to severe pathologies in humans also underline the essentiality of these ion channels as molecular sensors required for normal physiological function^38,51–54^. It has been confirmed that gain-of-function mutations in Piezo1 are linked to hereditary xerocytosis, while loss-of-function mutations induce lymphatic dysplasia^51–53^. Furthermore, mutations in Piezo2 cause distal arthrogryposis, Gordon syndrome and Marden-Walker syndrome^38,54^. Together, these mutant Piezo1/2 phenotypes provide novel insights into the mechanisms of related diseases.

#### Piezo1

Piezo1 channels allow mammalian cells to sense mechanical signals and consequently govern diverse physiological responses. Piezo1 senses shear stress and promotes vascular formation during the embryonic period; mouse embryos lacking Piezo1 die in the second trimester^55^. Furthermore, repressing the expression of the Piezo1 gene in neural stem cells suppressed neurogenesis but enhanced astrogenesis. Therefore, it was proposed that the Piezo1 channel determines the mechanosensitive lineage choice in neural stem cells^56^. The Piezo1 channel promotes T-cell activation by optimizing the process of TCRs recognizing MHC, which provides supportive evidence for the involvement of Piezo1 in human immunoregulation^57^. In addition, specifically knocking down Piezo1 in CD4^+^ T cells enhanced the proliferation of Tregs and consequently alleviated autoimmune encephalomyelitis in mice, which may be beneficial to the treatment of autoimmune diseases^58^. Moreover, other mechanobiological roles of Piezo1 require further investigation in other physiological processes, such as lymphatic development, red blood cell volume regulation, maintenance of intestinal and bone homeostasis, and jumping performance in humans^49,59–61^ (Table 1).

**Table 1:** Piezo1 channel in cellular mechanotransduction.

	Cells	Pathway and Function	References
Digestive System	Intestinal ECs	ROCK1/2→Claudin-1→Epithelial barrier function	^128^
	BC membranes	Ca^2+^transfer→BC contraction→Bile secretion	^129^
	Enterochromaffin cells	SSRNA → Piezo1 → 5HT release→Gut homeostasis	^49^
	Acinar cells	PLA2 → TRPV4 → Ca^2+^influx→Pancreatitis	^130,131^
	Mouse G cells	Antrum distension→Gastrin release→Control gastric activities	^132^
Musculoskeletal System	MLO-Y4 osteocytic cells	YAP1/TAZ → Wnt1→Bone anabolism	^133^
	IDG-SW3 osteocytic cells	Akt→Sost→Bone formation	^134^
	Mouse osteoblastic cells	**i**. Ca^2+^influx→AKT/GSK-3β/β-catenin→Runx2→Bone formation **ii**. Ca^2+^influx→ERK1/2/Perinuclear F-actin→Bone regeneration **iii**. YAP1 → COL2α1,COL9α2 → Bone anabolism	^135–138^
	Mouse liver ECs	Ca^2+^influx→PI3K-AKT/Notch→Angiogenesis in bone fracture healing	^139^
	UE7T-13 MSCs	BMP2 → Differentiation of MSCs→Bone homeostasis	^140^
	Mouse tenocytes	Ca^2+^influx→Collagen cross-linking→Tendon stiffness→Jumping performance	^61^
	Mouse primary adipocytes	FGF1/FGFR1 → Adipogenesis	^141^
Cardiovascular System	HUVECs	**i**. Vascular architecture and endothelial cell alignment **ii**. Ca^2+^influx/ATP release→P2Y2/G_q_/G_11_ → AKT/eNOS→NO release→Control blood pressure **iii**. Ca^2+^influx→Adrenomedullin release→cAMP→PKA/eNOS→NO release→Vascular tone and blood pressure **iv**. Ca^2+^/ATP → P2Y2/G_q_/G_11_ → FAK/NF-κB → Endothelial inflammation and atherosclerosis	^142–145^
	Mouse cardiomyocytes	Ca^2+^influx→Rac1/NOX2 → ROS → Heart homeostasis	^146^
	Mouse ECs	**i**. S1P → Piezo1/Ca^2+^influx→MT1-MMP → Angiogenesis **ii**. EDH(F) → Redistribution of blood flow **iii**. Embryonic development and vascular remodeling	^55,147,148^
	Human primary LECs	Lymphatic valve development and maintenance	^149^
	Zebrafish endothelial tip cells	Ca^2+^transients→Calpain/NOS → Brain vascular pathfinding	^150^
Blood System	Zebrafish RBCs	Erythrocyte volume homeostasis	^151^
	Mouse RBCs	Ca^2+^influx→KCa3.1 channel→Dehydration→Decreased cell volume	^60^
	Human RBCs	**i**. Ca^2+^influx/ATP release→Oxygen delivery, blood rheology, transfusion.etc. **ii**. Ca^2+^influx→Ca-ATPase→Increased glycolysis→RBC mechanical distortion	^152,153^
	Human platelets and megakaryocytes	Thrombogenesis	^154^
	Human erythroblast cells	Ca^2+^influx→NFATc2/EpoR→Erythropoiesis	^155^
Nervous and Endocrine System	Human neural stem/progenitor cells	Yap/Taz→Specification of neurons and gliocytes	^56^
	Xenopus RGCs	Axon growth in the brain	^156^
	Drosophila sensory neuron	CaMKII/NOS/PKG → Inhibition of axon regeneration	^157^
	Rat OPCs	CNS regeneration	^158^
	Rat β-cell lines	Ca^2+^influx→Insulin release	^159^
Immune System	Human T cells	Ca^2+^influx→F-actin scaffold→T cells activation	^57^
	Mouse BMDMs	**i**. AP-1 → EDN1 → HIF1α → Innate immune activation **ii**. NF-κB/STAT6 → IFNγ/LPS → IL4/IL13 → Proinflammatory and healing response **iii**. LPS → TLR4/Piezo1→CaMKII-Mst1/2-Rac1→Innate immune activation	^160–162^
	Mouse T_reg_ cells	TGFβ/SMAD → Restrained T_reg_ cells	^58^
	Mouse myeloid cells	HDAC2/Rb1→Myelopoiesis→Cancer and infectious disease	^163^
Respiratory System	Mouse lung ECs	**i**. Calpain→VE-cadherin/β-catenin/p120-catenin→Disassembly of AJs→Lung vascular hyperpermeability **ii**. Calpain→Src/VE-cadherin→AJs stabilization→Endothelial barrier homeostasis	^164,165^
	Rat ATI and ATII cells	Ca^2+^influx→ATP release from ATI cells→P2Y_2_ on ATII cells→Surfactant secretion	^166^
Reproductive System	Rat MUA ECs	Ca^2+^influx→NO, EDHF, prostacyclin release→Vasodilation during pregnancy	^167^
Sense Organs	Human trabecular meshwork cells	Ca^2+^influx→Focal adhesions→Intraocular pressure regulation	^168^
	Mouse trabecular meshwork cells and SC endothelial cells	Aqueous humor outflow	^169^

The Piezo1 channel plays an important role in mechanical transduction in different types of cells, although some have not been verified in humans. For example, in the digestive system, the Piezo1 channel is essential for intestinal epithelial barrier function, bile secretion, intestinal homeostasis, and gastric activity. In the musculoskeletal system, the Piezo1 channel is mainly involved in bone metabolism, bone formation, and bone regeneration. In the respiratory system, the Piezo1 channel regulates pulmonary vascular permeability, lung epithelial homeostasis, release of alveolar surfactant, and other physiological processes. Interestingly, an increase in pulmonary microvascular pressure caused by head trauma or high altitude will open the Piezo1 channel, leading to the degradation of VE-cadherin/β-catenin/p120-catenin protein, which in turn destroys AJs and decreases the lung endothelial barrier function. However, the opening of the Piezo1 channel caused by alveolar stretching will make AJs more stable and prevent the endothelial barrier from being destroyed. The author ascribed these opposing results to the difference in the type, direction, and magnitude of mechanical force.

#### Piezo2

The Piezo2 channel is a known indispensable factor in touch, proprioception, and tactile pain^62–64^, but recent studies have indicated its important role in regulating urination and maintaining skeletal integrity^65,66^. Humans with Piezo2 mutations showed abnormal voiding behaviors. Loss of Piezo2 expression in proprioceptive neurons led to deformity and dysplasia of the spine and hip joints, suggesting the crucial role of Piezo2 in maintaining skeletal integrity^66^. Knockout of the Piezo2 gene in tumor endothelial cells inhibited tumor growth by suppressing angiogenesis^67^. Additionally, Piezo2 in cochlear outer hair cells is an essential molecule for mediating ultrasonic hearing in mice, but it is not important for low-frequency hearing^68^. It was concluded that ultrasonic hearing and low-frequency hearing might use distinct auditory pathways (Table 2).

**Table 2:** Piezo2 channel in cellular mechanotransduction.

	Cells	Pathway and Function	References
Nervous System	Rat DRG neurons	**i**. GTP/Piezo2 → Increased RA current amplitude **ii**. Visceral sensation	^170,171^
	Mouse DRG neurons	**i**. Epac1/Piezo2 → Allodynia **ii**. Touch sensation **iii**. STOML3/cholesterol → Control the membrane mechanics → Sensitivity of Piezo2 → Tactile allodynia **iv**. Proprioception→Skeletal integrity **vi**. Mtmr2/PIP2 → Decreased RA current→Somatic sensation	^62–64,66,172–174^
	Mouse MTN neurons	Proprioception	^175^
	**i**. Mouse Merkel cells **ii**. Rat Merkel cells	**i**. Ca^2+^/AP → SAI in Aβ-afferent fibers → Touch sensation **ii**. Itch inhibition	^176–178^
	Mouse trigeminal ganglion neurons	Tactile pain	^179^
	Mouse baroreceptor neurons	Piezo1/2 control blood pressure	^180^
	Rat bone afferent neurons	Bone pain	^181^
Respiratory System	Mouse nodose sensory neurons and DRG neurons	**i**. Lung volume regulation and the Hering–Breuer reflex in adult mice **ii**. Lung expansion and efficient respiration in newborn mice	^182^
Digestive System	Mouse enterochromaffin cells	5-HT release and epithelial fluid secretion	^183,184^

The Piezo2 channel exerts physiological effects on the nervous system, respiratory system and digestive system. In the nervous system, the Piezo2 channel might be a key sensor for light touch, pain, and visceral sensation. The Piezo2 and Piezo1 channels of the baroreceptive nerve endings in the carotid sinus coordinate to control blood pressure. In addition, the Piezo2 channel is not only an indispensable sensor for proprioception but also important in maintaining the integrity of bones. Mice lacking Piezo2 will develop scoliosis and hip dysplasia. In the respiratory system, the Piezo2 channel is essential to establish effective breathing in newborn mice and maintain normal breathing in adult mice. The Piezo2 channel might set the mechanosensitivity of enterochromaffin cells and convert mechanical stimulation of the intestinal lumen into the release of serotonin.

## Roles of piezo channels in the urinary system

### Expression and distribution of Piezo channels

The urinary system is an essential human metabolic route and plays an essential role in maintaining homeostasis of the body’s internal environment. Piezo channels, as multipurpose mechanotransducers in mammals, govern physiological processes of the urinary system by sensing mechanical stimuli such as shear stress and bladder wall stretching^1,5^. Hence, it is indispensable to determine the expression and distribution of Piezo channels in the urinary system. Previous immunoblotting results on the lysates obtained from transgenic reporter mice revealed that Piezo1 was expressed in the following rank order: kidney> urethra> bladder> ureter^69^. Immunofluorescence studies showed that Piezo1 was predominantly expressed in glomerular endothelial cells, parietal cells of Bowman’s capsule, distal convoluted tubule endothelial cells, principal cells of collecting ducts and urothelial cells of the renal pelvis in the kidney^69^. Intriguingly, the conclusion of this study indicated that Piezo1 is almost undistributed in the proximal tubules, which is contrary to the study by Peyronnet R et al.^41,69^. It is still unclear whether antibody specificity gives rise to this discrepancy. Moreover, consistent with two other studies, Piezo1 was also expressed in the urothelial cells of the bladder and ureter, the interstitial cells of Cajal and smooth muscle cells but mainly in urothelial cells^69–71^. Furthermore, Piezo1 was found in the urethra and surrounding tissues, including stromal cells and striated muscle cells of the rhabdosphincter, vaginal epithelium, prostate gland, seminal vesicles and ejaculatory ducts^69^. Although various studies have comprehensively identified the distribution of Piezo1 in the mouse urinary system, its function has yet to be investigated. By comparison, Piezo2 in the mouse bladder is poorly understood. Marshall et al. reported that Piezo2 was expressed in 81.5% of CTB-labeled bladder afferent neurons and 74% of Krt20-positive umbrella cells, but their study used neither controls nor specific probes^65^. Another study suggested that Piezo2 was mostly limited to a scattered population of umbrella cells, even when using Piezo2-Cre-dependent lineage tracing^72^. Additionally, Piezo2 was detected in suburothelial fibroblasts, blood vessels and dorsal root ganglia cells^65,72^. Although the sites of Piezo2 expression in the bladder are controversial, a multitude of studies have investigated Piezo2-mediated mechanotranduction in bladder (patho)physiology.

### Physiological Roles of Piezo Channels in the Urinary System

#### Piezo1 Affects the Regulation of Urinary Osmolarity

The kidney regulates body fluid equilibrium by concentrating and diluting urine, which is the basis for maintaining a relatively stable internal physiological environment. For a long time, researchers have focused on polycystins in the kidney. Polycystins, present on the primary cilia of various cell types, such as renal epithelial cells and endothelial cells, are considered flow sensors and play a crucial role in the mechanosensory transduction of the kidney and blood vessels^73^. Nevertheless, achievements in scientific research on polycystins cannot wholly clarify the mechanobiology in the kidney. Fortunately, the discovery of Piezo channels provides researchers with a promising direction. Piezo1 is also a key mechanotransduction molecule that senses mechanical stimuli in the kidney and is important for regulating urinary osmolarity^74^. In adult mice, Piezo1 was preferentially expressed in the collecting duct principal cells of the inner medulla, while Piezo2 was minimally expressed^74^. Impaired urinary dilution and urea concentration after dehydration or fasting have been observed in mice with conditional renal epithelium Piezo1 KO^74^. A possible model is that during dehydration, Piezo1 on the medullary principal cells of the kidney might increase cAMP production by improving the activity of adenylyl cyclase-6, facilitating aquaporin-2 lipid membrane targeting to accelerate water reabsorption^74^. However, this hypothesis still needs to be verified by rigorous experiments. Animal models, such as zebrafish, which is the optimal model for studying kidney function, can be used to find their upstream and downstream targets^75^. Notably, polycystin-2 and its mutant PC2-740X can directly or indirectly interact with Piezo1 in renal tubular epithelial cells through their N-terminal domain to inhibit its mechanosensitivity, affecting the regulation of intrarenal pressure^42^. Together, these findings will provide a strong foundation for further studying kidney diseases related to increased intrarenal pressure.

#### Piezo is vital for micturition

**Fig. 3:**
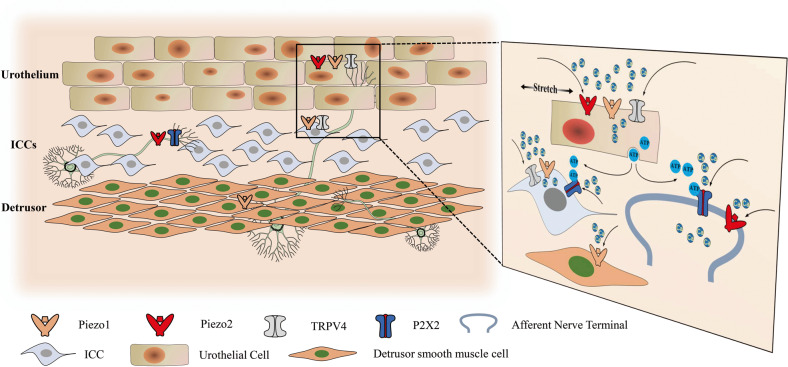
The hypothetical model of the Piezo channels participating in the micturition reflex. Under the mechanical stimulation of bladder wall stretching, MSC channels such as Piezo1/2 and TRPV4 of the umbrella cells are activated, and subsequently, Ca^2+^ flows into the cells, resulting in the release of ATP by exocytosis. Then, ATP binds to purinergic receptors on the afferent nerves of the bladder such as P2X2, thus transmitting urination control information to the central nervous system. The Piezo1 channel was also identified on the ICCs and detrusor smooth muscle cells, but their biological function in the urinary system is still unclear. The Piezo2 channel on the umbrella cells and the afferent nerves is considered to be the key to the initiation of the micturition reflex.

The bladder urothelium not only serves as a physical barrier but also functions as a “collector” of bladder urination signals, sensing mechanical bladder wall stretching, thereby causing Ca^2+^ influx and releasing adenosine triphosphate (ATP)^76,77^. ATP is the stimulant for the afferent nerves of the bladder. This molecule acts on P2X3 and other purinergic receptors on sensory neurons to communicate information regarding the degree of filling, initiate the micturition reflex and promote voiding^9,78,79^ (Fig. 3). In vitro experiments showed that Piezo1 is indispensable for primary cultured urothelial cells evoking Ca^2+^ influx and the release of ATP after sensing direct stretch stimulation^70^ (Fig. 3). Surprisingly, ATP release from the native urothelium was not dramatically reduced in conditional urothelial Piezo1 KO mice that also displayed normal voiding function and behavior^72^, suggesting potential compensatory mechanisms to overcome the loss of Piezo1. However, to date, there is still insufficient evidence to clarify whether Piezo2 overexpression can compensate for the loss of Piezo1^72^. Recently, a study reported that Piezo2 is a key mechanoreceptor for urinary function, renders the lower urinary tract sensitive to stretching, and initiates a duly timed micturition reflex in mouse models^65^. Marshall et al. monitored bladder pressure and sphincter activity in UPKII-cre+ Piezo2^fl/fl^ mice, whose Piezo2 in urothelial cells was widely deleted^65^. Knockout mice displayed a higher bladder stretch threshold, required higher bladder pressure to accomplish voiding and exhibited attenuated urethral reflexes^65^. However, these researchers used the same mouse strain to generate a urothelial Piezo2 KO model, and most of the studies used a small number of animals. Moreover, the researchers treated replicates as individual events instead of showing the mean/aggregate data for each individual animal. This method makes it unclear whether any of the results they show for cystometry or electromyography analysis are significantly different between the control and KO animals. Furthermore, they did not evaluate whether urothelial mechanotransduction and voiding behavior were affected in Piezo2 KO mice. Significantly, although Piezo2 KO mice and patients with Piezo2 deficiency reported voiding anomalies, their voiding function and behavior were not lost^65^. Dalghi et al. argued that conditional urothelial Piezo1 and Piezo2 KO mice exhibited near-normal mechanotransduction and voiding behavior^72^. In other words, knocking out urothelial Piezo1 or Piezo2 alone was insufficient, and instead, both channels must be knocked out simultaneously to reveal the true phenotype of urothelial Piezo channel-deficient mice. Specifically, the dual Piezo1/2 KO mice exhibited urinary incontinence during the active dark phase^72^. Thus, the role of Piezo1/2 in the lower urinary tract needs to be further studied. Moreover, the specific functional roles of Piezo1 in the urethra and detrusor remain largely unknown. Whether Piezo2 in blood vessels and fibroblasts also contributes to normal or abnormal LUT function deserves attention. Overall, Piezo1- and Piezo2-mediated ATP release in the urothelium is essential for normal urination function and behavior. These studies deepen our understanding of the connection between Piezo-dependent mechanotransduction and behavior, and more importantly, they lay the foundation for interrogating how urothelial Piezo1 and Piezo2 collaborate to control urination and determine how they affect abnormal LUT function.

The urinary function of the bladder is controlled by clock genes with circadian rhythms^80–82^. The chief function of the bladder during the day is to urinate and ensure regular and efficient urination frequency, whereas the urine storage function at night can facilitate better rest and sleep^83–85^. In the case of disruption of rhythmic homeostasis, diseases such as nocturia develop^86^. Recently, studies have claimed that the time-dependent gene expression pattern is applicable to Piezo1, as well as transient receptor potential vanilloid 4 (TRPV4), which is another well-known mechanosensitive ion channel^82,87,88^. Accordingly, the urothelium has a circadian rhythm of sensing bladder filling^89^. More specifically, the expression of Piezo1 and TRPV4 in the mouse urothelium increased during the active phase and decreased during the sleep phase^87^. Moreover, a mutation in the clock gene can keep the Piezo1 expression level constant in the bladder^87^. Consistently, in primary mouse urothelial cells, the oscillation of Ca^2+^ influx in response to Piezo1 and TRPV4 has a circadian rhythm, which might be a significant cause of circadian rhythm for the sensation of bladder filling^89^. Experimental evidence by chromatin immunoprecipitation suggested that the clock protein binds to the promoters of Piezo1 and TRPV4 to regulate transcription^87^. More critically, the role of GsMTx4 in lowering urine sensation in wild-type mice is related to the expression levels of the circadian clock and Piezo1^90^. The maximum effect is exerted during the sleep phase^90^. In this sense, reshaping the circadian rhythm of urinary function by inhibiting the Piezo1 channel might become a new treatment strategy for nocturia. However, it is worth noting that GsMTx4-mediated inhibition of the Piezo1 channel is nonspecific and affects other MSCs. Thus, we must account for this factor before drawing any conclusions that GsMTx4 benefits Piezo1-associated disorders.

### Pathophysiological roles of Piezo channels in the urinary system

#### Urological cancer

Piezo channels are directly involved in Ca^2+^ signal transduction or indirectly maintain the requisite electrochemical gradients for Ca^2+^ entry to affect the developmental process of tumors, including tumor cell proliferation, angiogenesis, migration, extracellular matrix remodeling, and the tumor microenvironment^91–94^. For instance, downregulating the expression of Piezo1 lowered intracellular Ca^2+^ levels in esophageal squamous cell carcinoma and then repressed tumor cell migration and invasion through the epithelial–mesenchymal transition pathway^95^. Knockout of Piezo1, which is highly expressed in gastric cancer tissues, significantly counteracted carcinogenic effects^96^. In addition, Piezo1 expression was upregulated in breast cancer, osteosarcoma, and glioma^97–99^. In contrast, there are few studies on the biological effects of Piezo2 in tumors. In gliomas, knocking down the expression of Piezo2 reduced the intracellular Ca^2+^ concentration, inhibited tumor angiogenesis and lowered vascular permeability through the Wnt/β-catenin pathway^67^. Thus, these discoveries have encouraged the study of the pathological roles of Piezo channels in urological tumors.

Over 90% of bladder cancer (BCa) occurs in the urothelium; this is the fourth most common malignant tumor in men^100^. In an experiment comparing the expression of Piezo between human and mouse BCa, real-time PCR (RT–PCR) analysis found that the mRNA expression of Piezo1 and Piezo2 increased significantly in both human BCa tissues and mouse BCa tissues^101^. Furthermore, the expression of Piezo1 is related to tumor stage, grade and size, while Piezo2 expression is correlated only with tumor stage^101^. Therefore, the activation of Piezo channels appears to contribute to the pathology of this condition. These studies initially examined the correlation between Piezo channels and BCa and provided a new direction for the investigation of BCa. Future studies will be needed to elucidate whether Piezo channels are associated with tumor cell growth and apoptosis, pyrolysis, autophagy, the immune microenvironment, and metabolism. Anatomically, the bladder urothelium is a layer of tightly connected cells covering the inner surface of the bladder and has a strong regenerative capability^102^. Understanding the cellular and molecular mechanisms underlying the homeostatic maintenance and repair of the urothelium is key to designing strategies against BCa. In zebrafish, Piezo1 senses cell crowding, promoting live cell extrusion to maintain homeostatic cell numbers in epithelia^103^. Gudipaty SA et al. also found that Piezo1 in epithelial cells recognizes two different mechanical signals, crowding and stretching^104^. Thus, Piezo1 controls cell division and apoptosis by affecting the intracellular Ca^2+^ concentration to modulate the number of epithelial cells in tissues and organs^104^. It can be inferred from these findings that Piezo1-mediated living cell extrusion might be a BCa suppressive mechanism that prevents the accumulation of excess epithelial cells. Moreover, Piezo1 might be required for urothelial repair and regeneration in BCa, and intensive mechanistic studies are urgently required to promote the scientific understanding of this issue. Piezo1 might be a promising target for the early prevention and management of BCa.

**Fig. 4:**
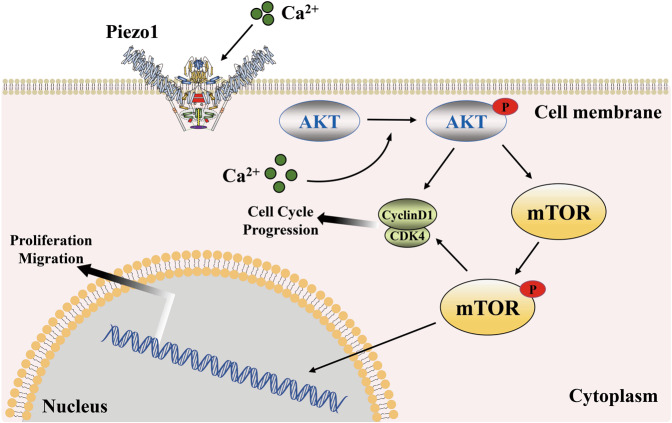
The Piezo1 channel promotes the tumorigenesis of prostate through the Akt/mTOR pathway (adapted from 111). The overexpression of Piezo1 might cause an intracellular Ca^2+^ increase, which directly or indirectly accelerates the phosphorylation of the AKT and mTOR. Phosphorylation of upstream targets might upregulate the expression of Cyclin D1 and CDK4 and promote the assembly of the Cyclin D1/CDK4 complex, contributing to the tumorigenesis of the prostate by promoting the cell cycle, as well as cell proliferation and growth.

Prostate cancer (PCa) is an epithelial malignant tumor that occurs in the prostate gland. Urinary system tumors have a relatively high morbidity and mortality rate, which seriously endangers the life and well-being of men^105^. PCa is related to a variety of signaling pathways, including the PI3K-AKT-mTOR/FOXO/NF-κB pathway, Wnt/β-catenin pathway, TGF-β/Smad pathway, and Notch signaling pathway^106–110^. Among these signaling pathways, the Akt/mTOR pathway has been shown to promote the development of PCa by its coupling with Piezo1^111^ (Fig. 4). Immunohistochemistry performed on 70 human PCa tissue specimens showed that the expression of Piezo1 was significantly upregulated, which was consistent with the results obtained by culturing PC3 and DU145 PCa cells in vitro^111^. A lentiviral vector expressing Piezo1 shRNA was applied to knock down Piezo1 in DU145 PCa cells. Then, the cells were injected subcutaneously to induce xenograft prostate tumors in immunodeficient mice^111^. It was found that the proliferation and migration of cancer cells in vivo were suppressed, suggesting a tumor-promoting role of Piezo1 in prostate tumors^111^. Further studies confirmed that repressing the expression of Piezo1 would impair intracellular Ca^2+^ signaling, hinder the phosphorylation of Akt and mTOR, and restrain the activation of cyclin D1 and CDK4, thereby inhibiting tumor growth^111^ (Fig. 4). However, most experiments have been based on cultured cells in vitro, which provides valuable information, and in vivo studies are lacking. Compared with the complex tumor microenvironment, artificially controlled conditions in vitro are suboptimal, and therefore, additional studies are required to investigate the consistency of the effects of Piezo1 in vivo and in vitro. In addition to immunocompromised animals, other immune-sound murine models should be considered for comprehensive evaluation of the role of Piezo1. Currently, the sole pathway identified for the mechanism of action of Piezo1 in prostate cancer progression is the Akt/mTOR pathway. Whether other pathways cooperate with Piezo channels to promote tumor progression remains unclear. Additionally, it cannot be discounted that cytosolic Ca^2+^, as the most abundant second messenger, plays a pivotal role in tumorigenesis, angiogenesis and metastasis^112^. The natural characteristic of preferential permeability to Ca^2+^ gives Piezo channels the potential to become biomarkers and new drug targets for the diagnosis and treatment of urological cancer. More investigations will focus on the tissue specificity of Piezo gene expression in bladder and prostate cancer and the downstream pathways stimulated by Piezo-mediated Ca^2+^ signaling.

#### Lower urinary tract dysfunction

Neurogenic and myogenic mechanisms can be used to explain overactive bladder (OAB). The neurogenic mechanism involves increased excitability of the central nervous system, and the sensitization of bladder afferent nerves causes dysfunctional bladder control^113,114^. The myogenic mechanism distinguishes OAB from unstable detrusor contraction^113,114^. In recent years, the urothelial-derived mechanism has gradually been accepted, opening up new horizons for the study of OAB. This theory states that the urothelium, acting as a nonneuronal interoceptor, improperly senses intravesical changes in pressure and chemistry, leading to OAB^115–119^. Accumulating evidence suggests that changes in the expression/sensitivity of urothelial mechanoreceptors might affect mechanosensitivity to stretching, resulting in abnormal bladder activity. For instance, the overexpression of TRPV4 on urothelial cells will increase the sensitivity of the urothelium to the sensation of bladder filling, inducing increased Ca^2+^ influx and subsequent excessive release of ATP^79,120^ (Fig. 3). With continuous intensive studies of Piezo channels, the urothelial-derived mechanism seems to be supported by credible evidence. Similar to TRPV4, mechanical stimuli, such as bladder wall stretching, evoke the Piezo1/2-mediated elevation of cytosolic Ca^2+^ and subsequent ATP release in the urothelium^70,72^ (Fig. 3). It has been reported that serosal ATP release is almost completely dependent on Piezo1/2-mediated mechanotransduction^72^. Furthermore, Piezo1 expression was significantly increased in the urothelium and ICCs of rats with cyclophosphamide-induced cystitis^121^. Urodynamic measurements or contractility tests of isolated rat bladder strips showed that GsMTx4 drastically alleviated bladder hyperactivity^121^. In addition, Piezo2-deficient patients displayed bladder underactivity. However, dual urothelial Piezo1/2 KO mice exhibited hyperactive/incontinent bladders rather than hypoactive bladders during an active dark phase^72^. Thus, the evidence seems to oppose the simple model that increased expression of urothelial Piezo1/2 could contribute to OAB. One possibility is that the biophysical properties of Piezo channels, such as inactivation characteristics, might have changed in the pathological state. Alternatively, discrepancies in these studies may reflect methodological approaches, antibody specificity issues, animal model problems and species differences. Overall, the mechanisms by which Piezos may affect OAB remain largely unknown but may be related to their role in urothelial mechanotransduction. Clearly, further research is needed to assess whether Piezo1/2-mediated mechanotransduction is affected in cases of hyperactive bladder. Notably, a previous report showed that specifically knocking down Piezo2 in sensory neurons in the mouse bladder extended the intervals between bladder contractions and increased the intravesical pressure required to initiate urination in murine models^65^, illuminating the downregulation of Piezo2 expression in bladder sensory neurons, which may be beneficial to the management of OAB. However, the understanding of the role of Piezo channels in bladder pathophysiology is far from comprehensive, and these promising results suggest that they are very appealing therapeutic targets for the treatment of bladder dysfunction.

#### Bladder outlet obstruction

Bladder outlet obstruction (BOO), defined from the perspective of clinical urodynamics, is difficulty in voiding. BOO is characterized by increased resistance of the urine outflow tract caused by a variety of pathogeneses, including benign prostatic hyperplasia in men, bladder neck obstruction in women and urethral stricture^122,123^. The clinical manifestations are mainly lower urinary tract symptoms, including storage symptoms (urination frequency, urgency and nocturia) and voiding symptoms (feelings of incomplete voiding, weak stream and hesitancy)^124^. Nevertheless, the pathological mechanism of bladder dysfunction caused by this obstructive disease has yet to be fully elucidated. Studying abnormal mechanotransduction in the lower urinary tract is a promising strategy. Bladder hyperactivity in mice with partial bladder outlet obstruction (pBOO) was linked to downregulated expression of the mechanosensitive TREK-1 potassium channel in the detrusor^125^. TRPV4 was associated with detrusor overactivity in rats with BOO^126^. Michishita M et al. produced a rat model of pBOO through partial urethral ligation and reported that Piezo1 mRNA in the suburothelium and detrusor increased observably on Day 7 after pBOO^71^. Moreover, a decrease in neurofilament expression with increased Piezo1 mRNA was observed^71^. This result suggests that Piezo1 participates in the compensatory mechanism of bladder denervation; however, the underlying molecular mechanisms remain unknown. Thus, evidence has implicated (although not verified) Piezo1 in detrusor–sphincter dyssynergia. BOO awaits further investigation, and additional evidence of Piezo1 involvement in the urethra and bladder outlet is needed before any conclusion can be drawn.

## Conclusions

In recent years, publications related to Piezo channels have shown a strong increasing trend^127^. This field has gradually become an intriguing research direction, deepening our understanding of mechanobiology in the urinary system. Considering the limited pharmacology of Piezo channels, it is still too early to discuss the underlying problems faced by their antagonists in clinical trials. However, the fact that inhibiting Piezo channels does not always induce beneficial results within the same tissue is unavoidable. One possible scenario is that Piezo1 might maintain urothelial homeostasis and prevent tumorigenesis by controlling the number of epithelial cells. In this situation, the applicability of Piezo1 antagonism in the management of bladder hyperactivity might be restricted. Although drugs that specifically target Piezo channels have a long way to go before reaching the clinic, we believe that studies on Piezo channels will undoubtedly change clinical practice to treat urinary system diseases.
